# Keeping up with the kids: the value of co-production in the study of irritability in youth depression and its underlying neural circuitry

**DOI:** 10.3389/fnbeh.2023.1124940

**Published:** 2023-06-15

**Authors:** Niamh MacSweeney, Perrine Louvet, Simal Zafar, Stella W. Y. Chan, Alex S. F. Kwong, Stephen M. Lawrie, Liana Romaniuk, Heather C. Whalley

**Affiliations:** ^1^Division of Psychiatry, Centre for Clinical Brain Sciences, University of Edinburgh, Edinburgh, United Kingdom; ^2^Department of Psychiatric Research, Diakonhjemmet Hospital, Oslo, Norway; ^3^PROMENTA Research Center, Department of Psychology, University of Oslo, Oslo, Norway; ^4^School of Life Sciences, University of Glasgow, Glasgow, United Kingdom; ^5^School of Psychology and Clinical Language Sciences, University of Reading, Reading, United Kingdom; ^6^MRC Integrative Epidemiology Unit at the University of Bristol, Bristol, United Kingdom; ^7^Population Health Sciences, Bristol Medical School, University of Bristol, Bristol, United Kingdom; ^8^Generation Scotland, Centre for Genomic and Experimental Medicine, Institute of Genetics and Molecular Medicine, University of Edinburgh, Edinburgh, United Kingdom

**Keywords:** irritability, adolescent depression, co-production, fMRI, adolescence

## Abstract

Irritability is a core symptom of adolescent depression, characterized by an increased proneness to anger or frustration. Irritability in youth is associated with future mental health problems and impaired social functioning, suggesting that it may be an early indicator of emotion regulation difficulties. Adolescence is a period during which behavior is significantly impacted by one’s environment. However, existing research on the neural basis of irritability typically use experimental paradigms that overlook the social context in which irritability occurs. Here, we bring together current findings on irritability in adolescent depression and the associated neurobiology and highlight directions for future research. Specifically, we emphasize the importance of co-produced research with young people as a means to improve the construct and ecological validity of research within the field. Ensuring that our research design and methodology accurately reflect to lives of young people today lays a strong foundation upon which to better understand adolescent depression and identify tractable targets for intervention.

## Introduction

Adolescence, a life phase spanning the ages 10–24 years, is a time of increased risk for the emergence of depressive disorders, which have a peak onset age of 19.5 years (Sawyer et al., 2018; Solmi et al., 2021). Importantly, rates of adolescent depression are rising. In the US, rates increased from 8.1% in 2009 to 15.8% in 2019 (Daly, 2022). Compared to adult-onset depression, adolescent-onset depression is associated with a more recurrent illness course and a host of physical and psycho-social difficulties with longer term consequences (Thapar et al., 2012; Malhi and Mann, 2018). It is therefore unsurprising that it is a leading cause of illness and disability for this age group (James et al., 2018). Taken together, these findings suggest that there are increasing unmet needs of adolescents with mental health difficulties (Wilson and Dumornay, 2022).

Unlike major depressive disorder (MDD) in adults, where low mood and anhedonia are primary diagnostic symptoms, irritability is considered an additional cardinal symptom specific to MDD in adolescence (American Psychiatric Association, 2013). Irritability can be defined as low frustration tolerance and an overreaction to blocked goal attainment relative to same-age peers (Stringaris et al., 2013; Avenevoli et al., 2015). While this can represent a normative behavior in adolescence, it becomes a pathological feature when associated with persistent functional impairment. Several behavioral studies demonstrate that high irritability in childhood and youth predicts later depression, suicidality, and impaired social functioning in adulthood (Leibenluft and Stoddard, 2013; Stringaris et al., 2013). This suggests that irritability may be an early indicator of emotion regulation difficulties and an actionable target for the prevention of downstream mental illness.

Existing definitions of irritability (i.e., proneness to anger/frustration) have shaped the primary experimental paradigms used in neuroimaging research on irritability, typically using frustrative non-reward and threat response tasks. Although these methodological approaches likely induce an irritable mood, the question remains as to whether they sufficiently tap into the broader social context in which the irritable mood occurs. This question is particularly pertinent when studying irritability in adolescence, a period during which mood and behavior are heavily impacted by one’s social environment (Blakemore and Mills, 2014; Sawyer et al., 2018). Here, we therefore seek to draw together current findings on adolescent irritability and its underlying neurobiology, not as a formal literature review (available elsewhere, see Nielsen et al., 2021 and Lee et al., 2022) but as a means to discuss opportunities for future directions in this field. Specifically, we highlight the value of co-produced research with young people as a way to ensure that our research design and methodology accurately reflect the lives of adolescents (MacSweeney et al., 2019; Whitmore and Mills, 2021). Improving the ecological validity of our research will help maximize the chances of identifying tractable targets for intervention that are appropriate for today’s youth.

## Defining irritability in the context of depression

According to current psychiatric nosology, irritability can be categorized as being chronic or episodic (Leibenluft et al., 2006). Chronic irritability in adolescence represents a young person’s baseline mood. Chronic irritability is considered a defining feature of disruptive mood dysregulation disorder (DMDD), whereby clinically significant irritability must have been present for at least 12 months (American Psychiatric Association, 2013). Conversely, episodic irritability, which refers to changes from baseline mood, is more often seen in mood disorders such as depression and bipolar disorder (American Psychiatric Association, 2013). The Diagnostic and Statistical Manual of Mental Disorders (Fifth Edition) (DSM-5) further distinguishes irritability into phasic and tonic. The former referring to behavioral outbursts of extreme anger from a high baseline, while the latter relates to angry mood lasting several days, weeks, or months. Although typically described in chronic irritability, phasic and tonic irritability may also be present within episodic irritability for the duration of the irritable mood (Vidal-Ribas and Stringaris, 2021). In terms of links between irritability and depression (see Vidal-Ribas and Stringaris, 2021 for further detail), the model with the most support is that of “shared risk factors.” That is, shared risk factors including genetic risk, family history of depression, temperament characteristics, and negative parenting styles, influence both outcomes (Vidal-Ribas and Stringaris, 2021).

## The neurobiology of irritability

While theoretical models of irritability can help contextualize factors associated with the emergence and development of irritability, identifying tractable targets for intervention requires an understanding of the neural mechanisms underpinning this salient and transdiagnostic marker of mental illness. The growing emphasis on adopting a translational neuroscience perspective is reflected in the significant increase in the number of studies published on the neural basis of irritability over the past decade (Nielsen et al., 2021; Lee et al., 2022). These studies have largely focused on exploring how irritability in childhood and adolescence, typically in clinical samples (e.g., youth with DMDD, attention deficit hyperactivity disorder (ADHD), internalizing difficulties), relates to changes in blood-oxygen-level-dependent (BOLD) signal, measured via functional magnetic resonance imaging (fMRI).

## Task fMRI studies

Task-based fMRI studies make up much of this relatively nascent field of research, which pivots upon three neurocognitive domains: threat processing/emotional reactivity, reward processing, and cognitive control. Threat processing and reward processing constitute the two brain/behavior pathways proposed by Brotman et al. (2017) in their translational neuroscience model of irritability (Brotman et al., 2017). Evidence for the threat processing pathway emerges from research which suggests that increased irritability is associated with an aberrant neural response in the medial (e.g., parahippocampal gyrus) and lateral (e.g., superior temporal gyrus) temporal regions, and the lateral prefrontal cortex (lPFC) (e.g., middle and inferior frontal gyri) when youth are presented with emotionally threatening stimuli, such as angry or fearful faces (Tseng et al., 2016; Wiggins et al., 2016; Kryza-Lacombe et al., 2020). Moreover, higher levels of irritability were found to be associated with more pronounced fluctuations in neural activation across task conditions (e.g., from congruent to incongruent trials), which may represent the additional effort required by youth with high irritability levels to process and respond to emotional stimuli in their environment. Despite the amygdala being the most commonly studied brain region in threat-processing studies on irritability, a recent review and meta-analysis by Lee et al. (2022) found that only 2 out of 12 studies reported increased amygdala activation in youth with higher levels of irritability (Lee et al., 2022).

Research supporting the reward processing pathway of irritability centers on frustrative non-reward tasks, whereby a frustrated psychological state is induced when the participant fails to receive a reward they have been conditioned to expect. Thus, the neural mechanisms of irritability are examined by inducing a frustrated state in real time and studying the associated neural correlates. Using this rigged reward paradigm, research has shown that youth with high irritability exhibit aberrant neural responses in fronto-striatal regions, such as the medial prefrontal cortex (mPFC), cingulate, caudate, and parietal regions, including the cuneus and precuneus, and inferior parietal gyrus, compared to typically developing youth (Deveney et al., 2013; Perlman et al., 2015; Tseng et al., 2019). Further, a recent study by Scheinost and colleagues used connectome-based predictive modeling and found that during frustration trials, functional connectivity within motor-sensory, subcortical and salience networks, and between these networks and fronto-parietal networks, was associated with increased levels of irritability (Scheinost et al., 2021).

**FIGURE 1:**
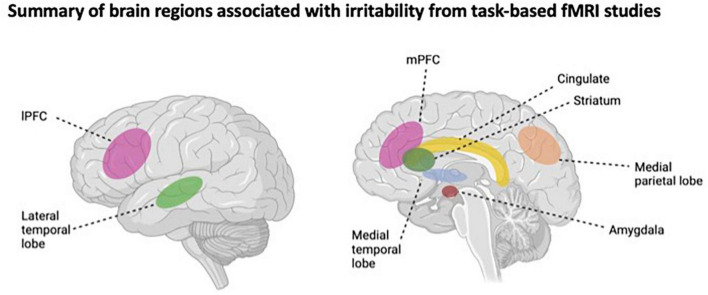
Illustrative summary of the brain regions associations with irritability from task-based fMRI studies. IPFC, lateral prefrontal cortex; mPFC, medial prefrontal cortex. This figure was made using BioRender.com.

Studies that have examined the neural basis of irritability via cognitive control fMRI tasks, such as inhibitory control paradigms (e.g., stop signal task, Flanker task), suggest that youth with high levels of irritability may exhibit inhibitory control deficits. For example, higher levels of irritability have been associated with aberrant patterns of neural activation in the superior temporal gyrus and pre- and post-central gyri (Chaarani et al., 2020), as well as the middle frontal gyrus, anterior cingulate, and striatum (Liuzzi et al., 2020). An illustrative summary of the brain regions associated with irritability in task-based fMRI research is shown in Figure 1.

Taken together, current paradigms used to examine irritability may be more likely to elicit fear or stress responses than more genuinely irritable ones, which highlights the need for tasks that aim to induce irritable mood specifically. Improving the construct validity of existing paradigms will have downstream effects on the ecological validity of the field by ensuring that our research methods capture the experience of youth irritability in the present day as accurately as possible.

## Resting state fMRI studies

**FIGURE 2:**
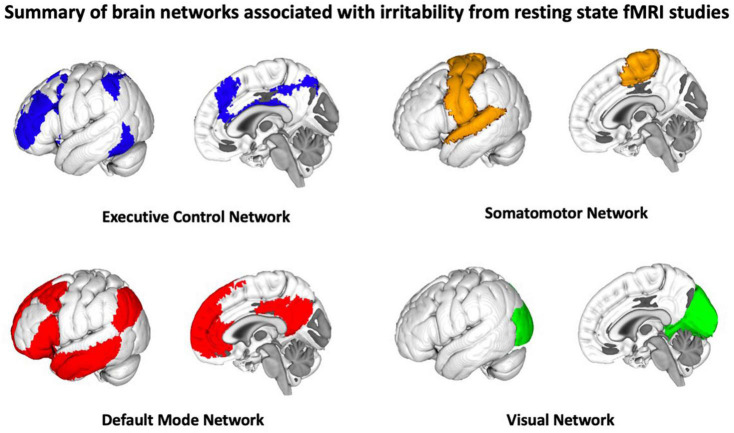
Summary of brain networks associated with irritability from resting state studies, the majority of which focus on the amygdala and its functional connectivity with other brain networks.

There are a limited number of resting state studies that have examined the neural correlates of irritability, the majority of which focus on the amygdala and its functional connectivity with other brain networks. In these studies, a questionnaire-based measure of irritability [e.g., the Affective Reactivity Index (ARI); Stringaris et al., 2012] is collected outside the scanner and then these behavioral measures are related to resting state imaging features. Most of the existing resting state studies focus on chronic irritability within the context of aggressive behavior/temper outbursts in childhood-onset disorders such as ADHD, oppositional defiant disorder (ODD), and autism spectrum disorder (ASD) (Bennett et al., 2017; Roy et al., 2018; Weathersby et al., 2019; Gaffrey et al., 2021), as well as some examining irritability in DMDD and bipolar disorder (Stoddard et al., 2015). Similar to the task-fMRI irritability literature, these studies suggest that the neural correlates of irritability comprise a diverse set of functional networks such as the default mode network (DMN), fronto-parietal network (FPN), executive control, sensory-motor, and visual networks (Nielsen et al., 2021). These networks support and coordinate cognitive processes associated with irritable mood, including self-referential behavior (DMN), reward processing and emotion regulation (FPN, executive control network), and motor response (sensory motor network). A summary of the brain networks associated with irritability are illustrated in Figure 2.

## Next steps for fMRI-irritability research

A recent systematic review and meta-analysis by Lee et al. (2022) sought to determine whether there are convergent neural responses associated with irritability across the domains of threat, reward processing, and cognitive control in task-fMRI (Lee et al., 2022). Although the authors conclude that, across individual task-based studies, the amygdala, putamen and caudate are the brain regions that exhibit the highest number of significant associations with irritable mood, they found little evidence for convergence across the three neurocognitive domains examined. For example, amygdala reactivity (increased activation) was found to be associated with irritability in only 2 out of 10 studies that used a threat-processing/emotional reactivity paradigm, 1 out of 3 studies that involved a cognitive control task, while no association was found in the two reward-processing task studies included in the review. The picture is equally unclear when considering the resting state literature on irritability. Current findings suggest that the neural correlates of irritability comprise amygdala functional connectivity with executive control, default mode and sensorimotor brain networks (Nielsen et al., 2021). However, the direction of these relationships was found to vary across individual studies with some reporting increased activation while others reported decreased or no activation (Nielsen et al., 2021). The inconsistent results across both task-based and resting state irritability studies may be due to a host of factors including, the marked heterogeneity in clinical characteristics, task design, irritability measures and analysis methods, small sample sizes, and a lack of longitudinal research. Moreover, it remains unclear whether the brain mechanisms underpinning irritability vary across disorders (Eshel and Leibenluft, 2020). Some evidence suggests that individual differences in dispositional (i.e., chronic) irritability may be more underpinned by amygdala-DMN connectivity than state (i.e., episodic) irritability, due to more consistent findings in this neural circuitry in resting state (Fulwiler et al., 2012; Gaffrey et al., 2021) compared to task-based studies (Stoddard et al., 2017; Kryza-Lacombe et al., 2020). However, more research, ideally combining resting state and task-based paradigms in larger samples with harmonized protocols is needed to advance our understanding of the neural correlates of irritability.

The heterogeneity present across multiple domains sheds light on several important considerations, especially research involving developmental samples. Probing individual differences in irritability will allow us to better understand its bounds as a normative behavior across development, and what might reflect concerning irritable mood. How this relates to other cognitive processes, such as emotion regulation, and the associated underlying neural circuitry, will help pave the way forward for targeted intervention strategies. Further, there is an overall paucity of research on age-related (and sex-related) changes associated with irritability — longitudinal and sufficiently powered cross-sectional studies that examine age interaction effects are also needed. It may be that the neural underpinnings of irritability vary across development and are related to other typical, as well as divergent, neurodevelopmental changes. Emerging research on brain growth charts for the human lifespan will be helpful in this effort (Bethlehem et al., 2022). Moreover, few studies have examined irritability in later adolescence (only 4/30 studies in the Lee et al. review had a mean age >15 years). Given the varying age of onset for mental health difficulties during adolescence (Solmi et al., 2021) and the distinct neuromaturation that characterizes this life phase (Bethlehem et al., 2022), future studies should be designed in a developmentally sensitive way. Some large, longitudinal youth cohort studies such as IMAGEN and the Adolescent Brain Cognitive Development (ABCD) Study, include variables related to irritable mood alongside neuroimaging data, and have already contributed to our understanding of the neurobiology of youth irritability and psychopathology (e.g., Chaarani et al., 2020, using IMAGEN data).

While the large sample sizes of such cohort studies are well powered to detect more subtle effects (e.g., individual differences in irritability, underlying neural circuitry, and potential contributing factors), the breadth of measures included in such studies comes at the cost of phenotypic depth. For example, ABCD and IMAGEN do not include an irritability-specific questionnaire like the ARI. Instead, a measure of irritability is derived from individual items in broad mental health measures like the Development and Wellbeing Assessment (DAWBA; Goodman et al., 2000) or the Child Behavior Checklist (CBCL; Achenbach, 2011). Thus, rather than a “panacea” to the many unknowns in developmental cognitive neuroscience, cohort studies may be better conceptualized as hypothesis generating tools that can inform directions for future studies (Saragosa-Harris et al., 2022). To develop a finer-grained characterization of irritability, and the functional significance of altered neural circuitry—especially how it relates to psychopathology—we need construct-specific and ecologically valid experimental designs. Ideally, these designs would involve harmonized protocols across studies to minimize sources of error as much as possible. Initiatives like the ENIGMA Irritability Working Group are leading by example in this way.

In sum, the surge of studies on the neurobiology of irritability over the past decade has allowed us to outline the brain areas involved in this transdiagnostic symptom from which myriad directions for future research have emerged. Before embarking upon these new avenues of research, we should reflect on *how* we plan to move forward to ensure that our journey takes us toward the world of young people rather than away from it.

## Discussion

### The value of co-produced research

Co-produced research, whereby the target population of the study, such as adolescents, are involved in as many steps of the research project as possible, has gained increasing traction in recent times.^1^ Initiatives like Young Persons’ Advisory Groups (YPAGs) allow young people to be involved in research in an active, meaningful, and mutually beneficial way. As co-researchers, young people and researchers can work together to ensure that the research questions, methods, and dissemination of research findings are relevant to the lives of young people today (MacSweeney et al., 2019). Although co-produced research involves a considerable (and front-loaded) time investment, researchers should approach it like other best practices in research, such as open science (Whitmore and Mills, 2021). Transparent and rigorous research that is attuned to the issues and experiences of today’s young people will be a key tool in our effort to answer complex questions in developmental science. Thankfully, resources are now available to help researchers undertake effective and meaningful co-created research (Whitmore and Mills, 2021).

### Toward a more ecologically valid study of irritability

Although there have been important commentaries on the neuroscience of irritability (Eshel and Leibenluft, 2020), the social context in which irritable mood occurs has been largely overlooked. Notably, irritability occurs in social and interactive contexts between youth and other people in their environment. However, existing fMRI paradigms, like frustrative non-reward tasks and emotional faces tasks, may not appropriately capture the rich social tapestry of adolescence. To enhance both construct and ecological validity, future research on irritability should incorporate social context into the study design. For example, Lee et al. (2022) propose a frustrative social non-reward task that targets behaviors like social rejection. This work would complement existing research on social exclusion in adolescence, which has used socially relevant tasks like Cyberball (Williams et al., 2000; Sebastian et al., 2010). Given that avoidance of social rejection drives adolescent decision-making and behavior (Tomova et al., 2021), this research could provide novel insight into how the nuances of the adolescent social world relate to the emergence and development of irritability and related mental health difficulties. Importantly, this effort to align our research methods with the social world of adolescence could be strengthened even further by undertaking research that is co-produced with young people.

By asking young people questions like, “*What situations do you find irritating in your daily life*?,” we could design studies that better reflect the experience of irritability as a young person. In turn, this could help disentangle the current heterogenous findings in irritability research. As mentioned by Lee et al. (2022), these insights could be incorporated into task-based fMRI, but there is also opportunity for “hybrid” resting-state paradigms. Recent calls for a “third-wave” of fMRI research propose the use of integrated fMRI paradigms, whereby task-like manipulations are paired with “traditional” resting state approaches (Finn, 2021). Naturalistic stimuli (e.g., movie watching) are some examples of integrated fMRI paradigms (Sonkusare et al., 2019), which allow researchers to regain some degree of experimental control, while acknowledging the dynamic patterns of brain function that arise from self-generated activity. Further, pairing these integrated paradigms with analyses capable of capturing fine grained temporal details, such as dynamic functional connectivity analysis, warrants consideration going forward. It has been argued that progress in our understanding of the human brain and behavior is likely to emerge from these “third-wave” paradigms (Finn, 2021). However, this progress will be hampered if the paradigms are not attuned to the lives of young people today. Co-produced fMRI paradigms will help ensure that the construct of interest is studied in a way that reflects real-world experience. For example, when studying youth irritability, we could ask young people to come up with irritating scenarios based on their own experiences. These scenarios would then form the stimuli for an integrated fMRI paradigm, asking young people to read each irritating scenario and imagine the experience as vividly as possible while in the scanner. This protocol would suit a range of samples (e.g., healthy volunteers, young people with mental health difficulties) but could also be adapted to suit different sample characteristics and research questions.

Importantly, novel paradigms like this would need to be validated against traditional task-based irritability paradigms (e.g., frustrative non-reward and threat response tasks) as well as behavioral measures of irritability (e.g., ARI). Given the lack of convergence in the neural correlates of irritability across neurocognitive domains (Lee et al., 2022), a novel, co-produced integrated fMRI task with improved ecological validity, holds great promise as way to better our understanding of youth irritability, identify tractable intervention targets, and move young people away from illness toward wellbeing.

## Author contributions

NM: conceptualization, methodology, formal analysis (literature review), investigation, writing—original draft, and writing—review and editing. PL: conceptualization, formal analysis (literature review), and writing—review and editing. SZ: conceptualization and writing—review and editing. SC: conceptualization, writing—review and editing, and funding acquisition. AK and SL: writing—review and editing. LR and HW: conceptualization, writing—review and editing, supervision, and funding acquisition. All authors contributed to the article and approved the submitted version.

## Footnotes

1
https://www.ukri.org/about-us/policies-standards-and-data/good-research-resource-hub/research-co-production/
